# Summer Excursions

**Published:** 1859-06

**Authors:** 


### HALL'S JOURNAL OF HEALTH.

OUR LEGITIMATE SCOPE IS ALMOST BOUNDLESS: FOR WHATEVER BEGETS PLEASURABLE AND HARMLESS FEELINGS, PROMOTES HEALTH; AND WHATEVER INDUCES DISAGREEABLE SENSATIONS, ENGENDERS DISEASE.

*We aim to show how Disease may be avoided, and that it is best, when sickness comes, to take no Medicine without consulting an educated Physician.*

VOL. VI.] JUNE, 1859. [No. 6.

### SUMMER EXCURSIONS.

In the beginning of June, "every body," except the most sensible few, and those who can afford to be independent and consult their own convenience in preference to fashion, begins to think of going somewhere; theoretically for health, really, to make observations, to plan alliances, to see if something or other won't turn up; or oftenest of all, in obedience to the behests of fashion. What an inexorable tyrant "every body does" is! More autocratic than any despot on earth; more remorseless than any man, beast or monster known; for there is no absurdity which it does not swallow; it halts at no sacrifice; at any moment, it is prepared so gulp down molten lead and swallow red hot shot, not only without wincing, but with a smirk or a grin, detestable tho' at best.

To all we say, get, in the first place, the requisite funds, after having paid the newsman, the milkman, the butcher, the grocer, the tailor and the dress maker the very last cent due; for summer is a hard time for them all, by reason of the general decrease of business, and how could you enjoy anything justly, with money which belongs to them?

With the first "quarter" of the surplus, purchase Dinsmore's Traveller's Guide, corrected at the first of each month, giving you an account of the times of starting of every "train," boat, steamship and stage in the union; the places to which they convey you; the distance, the time, and the expense. This will save you more bother of enquiry, than a dozen times the cost, to say nothing of the inconveniences arising from want of definiteness of information, and uncertainty of its reliability, with those provoking mistakes which are certain to occur at a cost of hours and days of time, and of dollars besides.

Determine next how long you can certainly be absent. Remove as far as possible all contingencies, which might require an earlier return than is desirable. The purse and "guide" and leisure being arranged, the manner of the performance is to be attended to.

If you are a "lone" man, under thirty, want a free and easy travel, and have a desire to see and learn a great deal, at the least possible expense of time, temper and money, have no baggage; leave overcoat, umbrella, overshoes, shawl, valise, every thing at home, take an extra shirt in one pocket, socks and handkerchief in the other, and go ahead, snapping your fingers at porters and hack drivers; all looking on you, while others are fuming and fretting for checks, or waiting for luggage, or bargaining for carriages, or quarreling with porters; always in a hurry; always in a fret, and always late; coming in, half the time, at the last tingle of the bell, or the drawing of the gangway, in such a swelter of heat, sweat and worryment, as to be the pity or the mirth of every looker on. The fact is, a large source of the amusement of travel, is the being able to witness the innumerable predicaments which the untravelled place themselves in, by their inexperience, thoughtlessness, or want of forecast. Having travelled in two hemispheres, we know whereof we affirm. Those who have traveled a great deal, and have learned very little, can testify that the disquietude about the baggage just before starting from one place, and just before, and on their arrival at another, absorbs a considerable part of their waking existence. We have repeatedty gone a hundred or two miles away, yes, a thousand, and as many more back, in the manner named above, and by a little management and attention, secured cleanliness and tidiness of dress all the time, by the intervention of rainy days and Sundays. It will pay in comfort, without much loss of money, if pressed for time, to throw away a soiled handkerchief, or collar, or sock, or drawer, and buy others, besides the fact that the gift of them to poor persons, would be quite a god-send.

If it is in contemplation to spend several days at a time in hotels in cities, or small towns, or to be a good deal in steamboats; to do so with the largest amount of comfort and complacence, in consequence of having the best things and the best places, the first and best and promptest attention from landlords, clerks, and servants, travel with a handsome woman. There is nothing like it. No fairy wand will "transmogrify" things so. Beauty "rules the roast" everywhere. It commands every body from hostler to host. You may yourself be a nobody; you may have a pug nose, a red head: you may be a perfect "duck" of a man; so short and fat, that you can't make even a respectable waddle; your face may be pock marked; your back may be humped; your shank a perfect spindle, and your leg, a bow—only have a magnificent woman along, and for her sake, you will be treated "all your journey through" as menials treat a master, as courtiers treat their king. We have tried it reader, in earlier years and later, and know its delights; not bothering ourselves with any over nice discriminations; comfort is comfort, whatever may be the motive from which it spring. A diamond is a diamond, although washed from the mud by a blackamoor

Our wives and daughters lose three-fourths of the pleasures of summer travel, by the inexcusable, the execrable perversion of true taste and common sense, in dressing for a rail car or a steamboat, as if they were going to a court reception. It does seem that they have no more sense of the fitness of things than idiots. Cannot some few gentlemen have their own way for once, and thereby set the fashion by dressing in the families for a summer travel in plain, substantial garments, allowing no member any thing beyond what a small carpet bag would contain, and which should be the sole article which each one was to take care of. Let us all "put ourselves upon our behavior," and not on our dress. The fact is, the clerks and proprietors of hotels, the captains of steamboats, and the conductors of railroads, see at the very first glance, the real *status* of a traveller; the dullest chambermaid, the most stupid cabin boy, and the laziest waiter, are neither dull, nor stupid nor lazy, nor erring either, in the estimate they make of people, as if by intuition. At "first sight," they don't see the dazzling watch chain; the fancy vest; the shiney hat; the point lace veil; the embroidered handkerchief; the cashmere shawl; the thousand dollar dress, and boundless crinoline; it is that presence which the face and features and countenance diffuse around, sustained by a gait, and manner, and modulation of voice, which the brightest of them could never describe, but which the meanest intellect of them all, cannot help but see and feel, at the instant of a first glance.

